# Cardiac deformation analysis from orthogonal CSPAMM (OCSPAMM) tagged MRI

**DOI:** 10.1186/1532-429X-14-S1-W17

**Published:** 2012-02-01

**Authors:** Hui Wang, Mo Kadbi, Motaz Alshaher, Melanie Kotys-Traughber, Stefan Fischer, Amir Amini

**Affiliations:** 1 University of Louisville, Louisville, KY, USA; 2 Philips Medical Systems, Cleveland, OH, USA

## Background

Magnetic resonance imaging (MRI) is a highly advanced and sophisticated imaging modality for cardiac motion assessment and quantitative analysis. The myocardial tagging techniques have seen wide applications for cardiac deformation analysis (see [1-4] for example). Among different image post-processing techniques, frequency-based methods have played an important role in analysis of heart displacement from tagged MRI images [5,6]. In this abstract, we apply the SinMod technique to data from a new tagging pulse sequence which we have recently developed called Orthogonal CSPAMM (OCSPAMM) [7].

## Methods

In OCSPAMM, the second SPAMM tag orientation is rotated 90 degrees relative to the first one so that motion information in two directions can be obtained simultaneously [7]. SinMod is a frequency-based method to analyze the heart deformation from tagged MRI [6]. In SinMod, the intensity distribution around each pixel is modeled as a cosine wave front. Both phase and frequency for each pixel are determined directly from the frequency analysis and the displacement is calculated from the ratio of phase difference and local frequency. The speed of SinMod method is as fast as HARP but SinMod has advantages in accuracy, noise reduction, and reduced artifacts.

## Results

**Figure 1:**
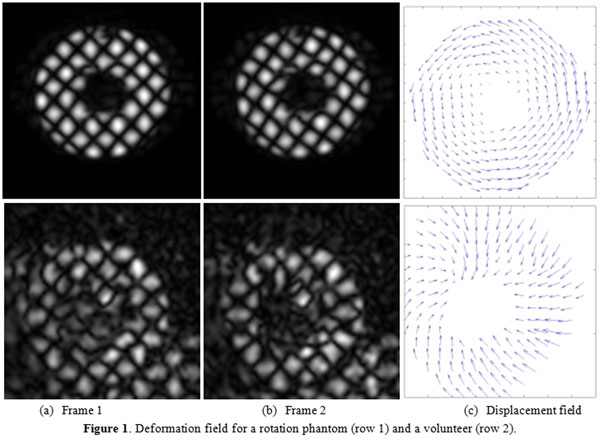


All tests were conducted on a 3T Achieva MR scanner (Philips Healthcare, Best, NL). A rotation motion phantom and a healthy volunteer were imaged using OCSPAMM. First row in Figure 1 shows two frames from the rotation phantom and the corresponding displacement field. The clockwise rotation can be observed clearly. Notice that as expected, the inner wall has less motion compared to the outer wall. The reason is that the inner wall is fixed with the axis and rotating force comes from the outer wall creating a shearing deformation. Second row in Figure 1 shows results in a healthy volunteer.

## Conclusions

We have proposed a new tagging sequence and cardiac deformation can be extracted from OCSPAMM tagged images. The images results showed good tag persistence and motion field verified the effectiveness of cardiac deformation analysis from OCSPAMM tagging technique.
